# ThermoSpots to Detect Hypothermia in Children with Severe Acute Malnutrition

**DOI:** 10.1371/journal.pone.0045823

**Published:** 2012-09-26

**Authors:** Thomas B. Mole, Neil Kennedy, Noel Ndoya, Alan Emond

**Affiliations:** 1 Centre for Child and Adolescent Health, University of Bristol, Bristol, United Kingdom; 2 Department of Paediatrics & Child Health, University of Malawi, Blantyre, Malawi; 3 Kamuzu College of Nursing, University of Malawi, Blantyre, Malawi; University of Barcelona, Spain

## Abstract

**Introduction:**

Hypothermia is a risk factor for increased mortality in children with severe acute malnutrition (SAM). Yet frequent temperature measurement remains unfeasible in under-resourced units in developing countries. ThermoSpot is a continuous temperature monitoring sticker designed originally for neonates. When applied to skin, its liquid crystals are designed to turn black with hypothermia and remain green with normothermia.

**Aims:**

To (i) estimate the diagnostic accuracy of ThermoSpots for detecting WHO-defined hypothermia (core temperature <35.5°C or peripheral temperature <35.0°C) in children with SAM and (ii) determine their acceptability amongst mothers.

**Methods:**

Children with SAM in a malnutrition unit in Malawi were enrolled during March-July 2010. The sensitivity and specificity of ThermoSpots were calculated by comparing the device colour against ‘gold standard’ rectal temperatures taken on admission and follow up peripheral temperatures taken until discharge. Guardians completed a questionnaire to assess acceptability.

**Results:**

Hypothermia was uncommon amongst the 162 children enrolled. ThermoSpot successfully detected the one rectal temperature and two peripheral temperatures recorded that met the WHO definition of hypothermia. Overall, 3/846 (0.35%) temperature measurements were in the WHO-defined hypothermia range. Interpreting the brown transition colour (between black and green) as hypothermia improved sensitivities. For milder hypothermia definitions, sensitivities declined (<35.4°C, 50.0%; <35.9°C, 39.2%). Specificity was consistently above 94%. From questionnaires, 40/43 (93%) mothers reported they were 90–100% happy with the device overall. Free-text answers revealed themes of “Skin Rashes”, “User-satisfaction” and “Empowerment".

**Conclusion:**

Although hypothermia was uncommon in this study, ThermoSpots successfully detected these episodes in malnourished children and were acceptable to mothers. Research in settings where hypothermia is common is needed to determine performance with certainty. Instructing users to act when the device’s transition colour appears could improve accuracy. If reliable, ThermoSpots may offer simple, acceptable and continuous temperature measurement for high-burden areas and reduce the workload of over-stretched staff.

## Introduction

Severe acute malnutrition (SAM) is responsible for approximately 1 million child deaths per year worldwide [1]. Hypothermia is a recognised serious complication of SAM [2], [3] and is a risk factor associated with increased mortality [4], [5]. In an attempt to conserve calories, the metabolic rate is reduced, leading to an inability to generate sufficient heat. Hypoglycaemia and infection, both associated with malnourished children [3], increase the risk of hypothermia [6]. Malnourished children also lose heat more rapidly than well-nourished children as they have less insulating body fat and an increased body surface area to weight ratio [7]. Hypothermia further effects malnourished children through reduced cardiac output and reduced cerebral perfusion [6].

The World Health Organisation (WHO) recommends regular 2–4 hourly temperature measurements of children being treated for SAM, while they are in the ‘stabilisation phase’ of treatment [8]. Yet in developing countries, regular temperature monitoring remains a challenge [5]. A single temperature measurement may require a minute of a health professional’s time [5]. As paediatric wards in resource-poor settings are understaffed and overstretched, such monitoring can become unfeasible. Consequently, hypothermia often remains undetected, undocumented and untreated [9].

**Figure 1:**
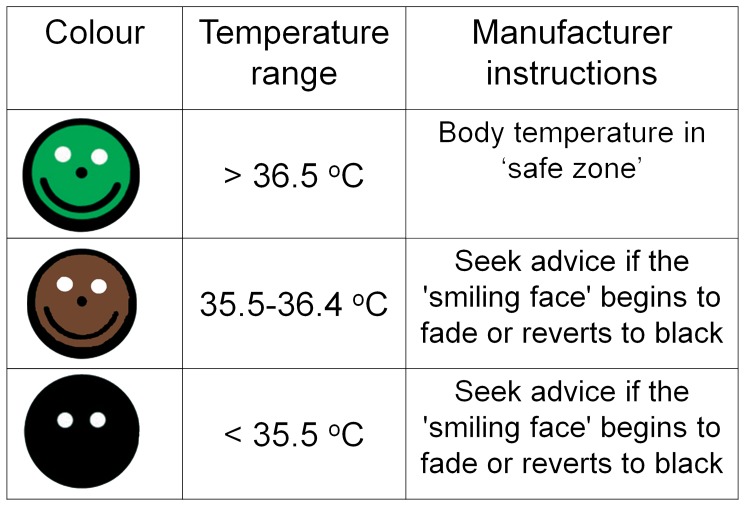
ThermoSpot’s liquid crystal colour profile.

The ‘ThermoSpot’ was developed as a simple, inexpensive means of detecting hypothermia in neonates. It is an adhesive 12 mm sticker that is stuck onto a baby’s skin. A liquid crystal compound embedded in the upper surface changes colour with temperature. According to the manufacturer’s documentation supplied at the time of the study [10], when used in the axilla or upper abdomen, below a peripheral temperature of 35.5°C the liquid crystal disc is black with two white dots in the upper half (accuracy±0.5°C) (figure 1). According to the WHO [8], this corresponds to a core temperature of approximately 36°C (core temperatures being approximately 0.5°C warmer than peripheral temperatures). Between 36.5 and 37.5°C (core temperature 37–38°C) a “smiling face will appear on a bright green background”. In the transition zone between 35.5 and 36.4°C (core temperature 36–36.9°C), the manufacturer’s original documentation states “the background colour will begin to fade to … a ‘terracotta’ colour” or turn brown. Carers are taught to check the ThermoSpot colour and wrap up the child, initiate ‘Kangaroo care’ [8] and inform nursing staff if it turns black.

When evaluating a device designed to detect hypothermia, it is the sensitivity rather than specificity of the device that is most important. Although various definitions of hypothermia have been used, ThermoSpots have been shown to be highly sensitive (88–100%) in both community and hospital settings for detecting significant hypothermia in neonates [11], [12]. ThermoSpots have also been used successfully with illiterate carers [13]. These results have been obtained with the device applied to the axilla or epigastrium.

However the use of ThermoSpots has not been assessed in children with SAM. It is not known how ThermoSpots perform over the manufacturer’s stated temperature thresholds and within WHO definitions of hypothermia in children with SAM – which are different from those in neonates. In addition, it is unclear if continuous ThermoSpot thermometry is acceptable to parents. So far, perceptions of ThermoSpots have been limited to comments and observations from providers [13] rather than users.

The aims of this study were to: (i) estimate the diagnostic accuracy of ThermoSpots for detecting WHO-defined hypothermia (core temperature <35.5°C or peripheral temperature <35.0°C) in children with SAM and (ii) determine their acceptability amongst mothers.

## Methods

### Ethics Statement

Ethical approval was obtained from the College of Medicine, University of Malawi in accordance with the Helsinki Declaration. The study received no external funding. Mr John Zeal of Camborne Consultants donated the ThermoSpots necessary for the study free of charge. Mothers gave informed written consent.

### Study Setting and Subjects

Consecutive children with SAM were enrolled from the malnutrition ward of Queen Elizabeth Central Hospital, Blantyre, Malawi (longitude 15°80′S, Latitude 35°02′E) from March-July 2010– including the coldest months of the year. Inclusion criteria were children over the age of 1 month with SAM, that met the WHO’s definition [14]. This definition was based on National Centre for Health Statistics weight-for-height criteria. Children with severe skin problems that prevented ThermoSpot adherence were excluded.

### Accuracy

The ThermoSpot was placed on the child’s supraclavicular fossa as this avoided the need to remove clothing and provided the mother with an accessible site to monitor temperature. Using a digital electronic thermometer (DET) accurate to 0.1°C (compliant with ASTM industry standard specification E1112), the initial rectal temperature (the gold standard core temperature) was compared with the ThermoSpot colour. The ThermoSpot remained in place for the remainder of the child’s admission. Subsequent follow-up axillary DET measurements were routinely taken by nurses up to 4 times a day, depending on staff availability, and the colour of the ThermoSpot was recorded. All DET measurements were recorded to one decimal place in degrees Celsius. Data were collected until the patient was discharged or died. Posters were used to ensure all enrolled mothers and nurses used ThermoSpots consistently and responded to hypothermia by initiating appropriate thermal care. To minimise inconsistencies from inter-observer variation and evaluate the effect this may have on detection rates, ThermoSpot’s colour was recorded as black, green, or ‘brown’ when it was neither clearly green nor obviously black. The ThermoSpot was replaced if it fell off and was reapplied with 3 M Transpore transparent surgical tape.

Throughout the study, the WHO definition [8] of hypothermia in SAM was used (core temperature <35.5°C or peripheral temperature <35.0°C).

The sensitivity and specificity of the ThermoSpot was calculated against DET measurements over a range of core temperatures from 35 to above 36.4°C. In particular, we evaluated the performance of the device to detect a peripheral temperature of less than 35.5°C – the temperature at which the manufacturer states it will turn black.

To achieve a sensitivity and specificity of over 95% with a confidence interval of less than 5%, the required sample size was calculated as 140 participants with one initial rectal measurement compared with one ThermoSpot measurement per participant.

### Acceptability

In the final month of the study, mothers of enrolled children were offered questionnaires. Usability, clarity, simplicity and overall user-satisfaction were assessed using visual Likert scales. Free-text questions to capture the qualitative views of mothers were also used. Mothers’ answers were transcribed immediately and translated by a native Chichewa speaker. Thematic analysis of free-text responses was performed by the principal investigator and validated by a local student nurse.

## Results

**Figure 2:**
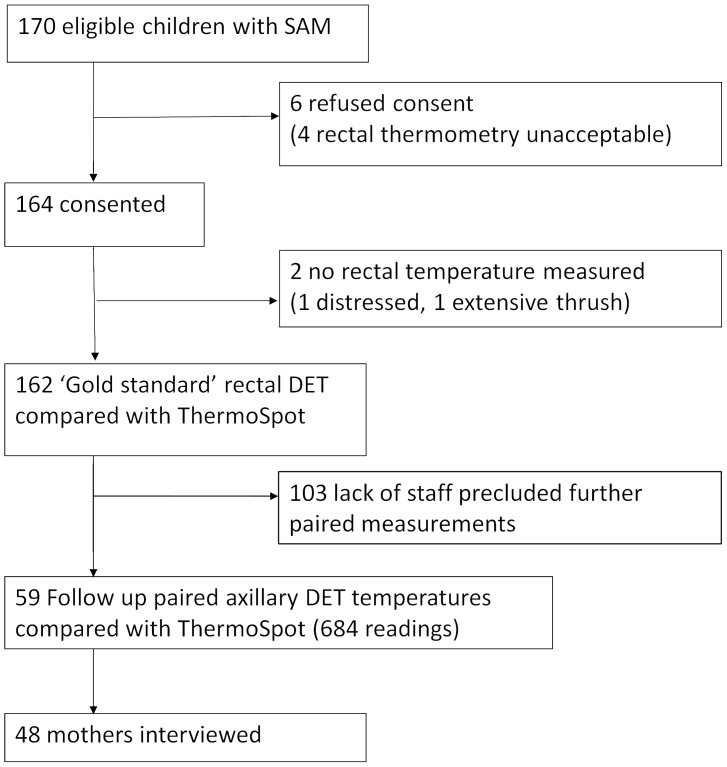
Recruitment and patient flow.

Of the 170 participants invited to enrol, 164 gave consent (Table 1, figure 2). The ThermoSpot was compared with rectal DET in 162 participants. Availability of nursing staff allowed follow-up axillary DET measurements to be compared with ThermoSpot in 59 participants. Of these, 43 mothers were available to be offered questionnaires, all of whom responded.

**Table 1:** Baseline demographic characteristics.

Category		Result
Mean age in years		2.1 (SD 1.6)
Gender	Male	88 (54%)
	Female	74 (46%)
Type of malnutrition	Kwashiorkor	79 (49%)
	Marasmus	71 (44%)
	Marasmic Kwashiorkor	12 (7%)

### Incidence of Hypothermia

Hypothermia was uncommon. Of 162 initial rectal measurements, only one met the WHO definition of hypothermia in SAM (core temperature <35.5°C). On follow up axillary measurements, two episodes of hypothermia occurred (peripheral temperature <35.0°C) out of 684 readings. Overall, 0.35% of readings from both core and peripheral temperatures were in the hypothermic range. No serious adverse events related to the use of ThermoSpot occurred, although skin rashes were reported (see Acceptability).

### Accuracy

The colour of the ThermoSpot was compared with the gold standard of DET rectal thermometry in 162 children, with one measurement taken per child (table 2).

**Table 2:** Rectal DET measurement compared to ThermoSpot colour.

ThermoSpot colour	‘Gold standard’ rectal (core) temperature (°C)				Total
	≤35.4*	35.5–35.9	36.0–36.4	≥36.5	
Green	0	6	20	132	158
Brown	0	0	1	1	2
Black	1	1	0	0	2
**Total**	1	7	21	133	162

* WHO definition of core hypothermia in SAM.

The performance (sensitivity and specificity) of the ThermoSpot versus rectal DET for a range of definitions of hypothermia is shown in table 3. The performance was calculated for both interpretations of the brown transition colour (normothermia or hypothermia).

**Table 3:** Performance of ThermoSpot compared to rectal DET for a range of definitions of hypothermia.

ThermoSpot performance	Definition of hypothermia (core rectal temperature in °C)		
**‘Brown’ interpreted as normothermia:**	**≤35.4** *	**≤35.9**	**≤36.4**
Sensitivity %	100	25	7.4
Specificity %	99.4	100	100
**‘Brown’ interpreted as hypothermia:**			
Sensitivity %	100	25	10.3
Specificity %	98.1	98.7	99.2

* WHO definition of core hypothermia in SAM.

The ThermoSpot detected the one occasion when the rectal temperature was less than 35.5° (the WHO definition of hypothermia). It did not turn black on 6 of 8 occasions when the core temperature was under 36°C - a sensitivity of 25% for this definition of hypothermia.

The comparison between the subsequent axillary measurements and ThermoSpot is shown in tables 4 and 5 (n = 684 measurements in 59 children).

**Table 4:** Axillary DET measurement compared to ThermoSpot colour.

ThermoSpot colour	Axillary (peripheral) temperature (°C)				Total
	≤34.9*	35.0–35.4†	35.5–35.9	≥36.0	
Green	0	8	26	610	644
Brown	1	4	13	14	32
Black	1	2	1	4	8
**Total**	2	14	40	628	684

* WHO definition of peripheral hypothermia in SAM.

† Peripheral temperature at which manufacturer states device will indicate hypothermia.

**Table 5:** Performance of ThermoSpot compared to axillary DET for a range of definitions of hypothermia.

ThermoSpot performance	Definition of hypothermia (peripheral temperature in °C)		
	≤34.9*	≤35.4†	≤35.9
**‘Brown’ interpreted as normothermia:**			
Sensitivity %	50	18.8	5.7
Specificity %	99.0	99.3	99.3
**‘Brown’ interpreted as hypothermia:**			
Sensitivity %	100	50	39.2
Specificity %	94.4	95.2	97.1

* WHO definition of peripheral hypothermia in SAM.

† Peripheral temperature at which manufacturer states device will indicate hypothermia.

An axillary peripheral temperature under 34.9°C (the WHO definition of hypothermia) was recorded on two occasions. The corresponding ThermoSpot colour was black on one occasion and brown on the other. Interpreting the brown transition colour as hypothermia improved the sensitivity of the device for all three axillary temperature ranges. Specificity was consistently above 94%. The ThermoSpot remained green on 8 of 16 occasions (50% sensitivity) when the temperature was less than 35.4°C – the temperature at which the manufacturer states the device will indicate hypothermia.

No potentially serious adverse events of a ThermoSpot incorrectly indicating hypothermia in the presence of hyperthermia occurred.

### Acceptability

All 43 mothers who were approached for interview consented. When asked “How clear was the colour on the ThermoSpot?”, 40/43 (93%) mothers reported from Likert scales that they were 90–100% happy with the device overall. Similarly, when asked “How happy were you with the device overall?”, 40/43 (93%) mothers agreed the device was 90–100% clear.

Lastly, mothers were asked “Do you have any other comments or feedback on the device?”. Three themes emerged from these free-text responses: user-satisfaction, empowerment and rashes.

#### User-satisfaction

32/43 (74%) of mothers’ free-text comments expressed high user-satisfaction, which included:

“I like it”

“It’s very good!”

“I am very happy [with the ThermoSpot]”

When applied to the child’s supraclavicular fossa, ThermoSpots were perceived as simple and beneficial. Having the ThermoSpot easily visible provided reassurance:

“It is good because you can easily know the condition of your baby.”

“I see the green colour and this is nice.”

“It helps us to know how our child feels.”

Mothers believed the device to be useful and effective:

“The device is helpful… All we have to do is continue to wrap up our babies.”

“It has really helped my son”

#### Empowerment

In 16/43 (38%) of comments, mothers reported being empowered to initiate thermal care without having to rely on a health professional:

“This machine assists me… it helps me know what to do”

“It helped me know how my baby’s body temperature is at all times”

“It is good to use this machine…whether the doctor is there or not, we know how to monitor the temperature personally”

18 mothers were asked if they had checked and noticed brown or black colour changes (not during routine paired-measurements) within the previous 24 hours. 14 reported at least one occurrence despite all routine recorded temperature measurements being normothermic. All mothers subsequently initiated appropriate thermal care.

#### Skin Rashes

Rashes caused by ThermoSpot were reported in 7/43 (16%) of mothers. This only occurred when transparent tape had been reapplied to a non-adherent ThermoSpot. Despite the rashes, mothers were still willing to use ThermoSpots:

“It did make the child have a rash, even so, everything is alright”

“The machine can cause rashes, but still the machine is good”

“I see no problem but the machine causes some rashes”

## Discussion

In this study of children with SAM, ThermoSpots successfully detected hypothermic episodes as defined by the WHO (core temperature <35.5°C, peripheral temperature <35.0°C) and were perceived positively by mothers. However, such significant hypothermia was uncommon and the calculated sensitivities were based on a limited sample of only three hypothermic episodes. In addition, the device failed to operate within the parameters described by the manufacturer and detect less severe hypothermia.

The device detected the one episode when the core temperature fell below 35.5°C. If the brown transition colour is interpreted as hypothermia, both hypothermic episodes below a peripheral axillary temperature of 35.0°C were also detected. Based on these few measurements, ThermoSpot’s sensitivity in detecting hypothermia in children with SAM may be satisfactory.

However, we found that although the manufacturer claims the device will turn black below a peripheral temperature of 35.5°C, for a core temperature of less than 36.0°C (approximately equivalent to a peripheral temperature of 35.5°C), ThermoSpot detected only two of the eight episodes of hypothermia. In addition, despite mothers finding the ThermoSpots straightforward, interpreting the transition colour change was somewhat subjective. When the ‘smiling face’ begins to appear, mothers may read the brown colour as normothermia. Interpreting it like this reduced the sensitivity from 50.0% to 18.8% for peripheral temperatures below 35.5°C – the manufacturers specification. This ambiguity over the colour change reduces the reliability of the device over its specified temperature range and reduces its accuracy in detecting less severe hypothermia, though this may not be of clinical significance for children with SAM. A potential limitation of our study was that we compared multiple measurements (total 684) of peripheral temperature against ThermoSpot colour in 59 children thus introducing a possibility of bias. That is, we may inadvertently have been repeatedly assessing the performance of the device in children in whom the ThermoSpot performed particularly well or badly. However the sample size for the single paired comparison of rectal temperature versus ThermoSpot was exceeded, and as the results in this group are similar to those in the peripheral temperature group, we think that the peripheral temperature results are highly likely to be valid.

This lower performance for less-severe hypothermia could be explained by the site we chose to place it – the supraclavicular fossa. Previous studies (in neonates) have used the axilla or epigastrium. However these sites were deemed unsuitable in children with SAM as reading the ThermoSpot would necessitate removing the child’s clothing, perhaps predisposing them to hypothermia. This type of continuous monitoring device needs to be easily accessible.

Alternatively, could the physiological and size differences between neonates and children with SAM have affected its performance? This appears to be unlikely. Although our results were obtained in older children, our findings are similar to those of a large neonatal study which reported a sensitivity of 18% in the 32–36°C range of peripheral temperatures [15].

Despite conducting the study during the coldest times of the year, the low incidence of hypothermia was striking. One explanation for the low levels of hypothermia is that this urban paediatric ward was well relatively well-equipped and staffed for the region. In addition, mothers on the ward shared beds with their children, which may have improved thermal care. It is also possible that there could be more cases of uncomplicated SAM than expected in our inpatient sample, as there is no option for a direct referral for community-based care at our hospital. This could partly explain the lower than expected prevalence of hypothermia. Alternatively, significant hypothermia in SAM may not be as common in tropical settings as previously described [2]. Previous studies from Kenya also report low rates of hypothermia in low-resource settings [5]. This study adds weight to the argument that given low hypothermia burdens, the WHO’s guidance of 4–hourly temperature monitoring may not be necessary and more targeted temperature monitoring may be more appropriate in some settings [5]. Incidentally, although 3 hypothermic episodes were detected by DET, 14/18 mothers reported hypothermia within the previous 24 hours. This shows that the recorded incidence was lower using DET than ThermoSpot – though this study was not designed to determine the incidence of hypothermia.

Previous published comments from health providers have suggested that ThermoSpots are acceptable to mothers. Despite the recognised need to use methods of preventing hypothermia that are ‘user-friendly’ [16], these assumptions have remained largely untested. This research provides original evidence to confirm these suppositions. ThermoSpot’s acceptability for illiterate mothers was demonstrated, and the previously undocumented benefit of maternal empowerment was revealed. Mothers appreciated the opportunity to provide better care for their sick child and recognised their ability to reduce reliance on overstretched staff. This suggests that if ThermoSpots are recommended for wider use, uptake and compliance rates could be favourable. Given ThermoSpot’s intuitive way of demonstrating the importance of hypothermia [17], liquid crystal thermometry could also play an important role in delivering health education to improve awareness and change health behaviours relating to thermal care.

The only adverse affect detected was the presence of a rash in children that needed some medical adhesive tape to keep it from falling off. However, most mothers did not regard the rash as a significant problem.

ThermoSpots currently cost US$0.14 per device. In our study, most ThermoSpots needed replacing within a week. If introduced for universal use in our unit, we estimate an additional annual cost of approximately $550. This is not insubstantial in a poorly resourced environment.

### Conclusion

ThermoSpots appear to be an acceptable method of detecting WHO-defined hypothermia in children with SAM. However, further research in a setting where hypothermia is more common is required to conclusively evaluate their performance across a range of temperatures. Instructing users to act when the device’s transition colour appears could improve accuracy. Cost-benefit studies to take into account different levels of disease burden from hypothermia are also needed. In our low-prevalence setting, we would not at present recommend the routine use of ThermoSpots. However, if used in high-burden contexts, with optimal colour-change temperature thresholds, ThermoSpots could offer simple, acceptable and continuous temperature measurement that could reduce the demand on overstretched medical staff.
